# Viral Diversity of House Mice in New York City

**DOI:** 10.1128/mBio.01354-17

**Published:** 2018-04-17

**Authors:** Simon H. Williams, Xiaoyu Che, Joel A. Garcia, John D. Klena, Bohyun Lee, Dorothy Muller, Werner Ulrich, Robert M. Corrigan, Stuart Nichol, Komal Jain, W. Ian Lipkin

**Affiliations:** a Center for Infection and Immunity, Columbia University, New York, New York, USA; b Viral Special Pathogens Branch, Centers for Disease Control and Prevention, Atlanta, Georgia, USA; c Chair of Ecology and Biogeography, Nicolaus Copernicus University, Toruń, Poland; d RMC Pest Management Consulting, Briarcliff Manor, New York, USA; Vanderbilt University Medical Center

**Keywords:** environmental microbiology, microbial ecology, microbial genetics, New York City, veterinary microbiology, mouse virome, viral diversity

## Abstract

The microbiome of wild *Mus musculus* (house mouse), a globally distributed invasive pest that resides in close contact with humans in urban centers, is largely unexplored. Here, we report analysis of the fecal virome of house mice in residential buildings in New York City, NY. Mice were collected at seven sites in Manhattan, Queens, Brooklyn, and the Bronx over a period of 1 year. Unbiased high-throughput sequencing of feces revealed 36 viruses from 18 families and 21 genera, including at least 6 novel viruses and 3 novel genera. A representative screen of 15 viruses by PCR confirmed the presence of 13 of these viruses in liver. We identified an uneven distribution of diversity, with several viruses being associated with specific locations. Higher mouse weight was associated with an increase in the number of viruses detected per mouse, after adjusting for site, sex, and length. We found neither genetic footprints to known human viral pathogens nor antibodies to lymphocytic choriomeningitis virus.

## IMPORTANCE

Mice carry a wide range of infectious agents with zoonotic potential. Their proximity to humans in the built environment is therefore a concern for public health. Laboratory mice are also the most common experimental model for investigating the pathobiology of infectious diseases. In this survey of mice trapped in multiple locations within New York City over a period of 1 year, we found a diverse collection of viruses that includes some previously not associated with house mice and others that appear to be novel. Although we found no known human pathogens, our findings provide insights into viral ecology and may yield models that have utility for clinical microbiology.

## INTRODUCTION

Wild *Mus musculus* (house mouse) is an adept colonizer of the built environment and an important rodent pest species. House mice have been associated with the transmission of two zoonotic agents, *Leptospira* spp. (1) and lymphocytic choriomeningitis virus (LCMV) (2); both are transmitted through contact with murine excreta. The carriage of other pathogenic organisms, such as *Enterococcus faecium* (3), *Clostridium difficile* (4), and *Salmonella* spp. (5), has also been demonstrated, further illustrating their potential to act as a zoonotic reservoir. Serosurveys conducted in Baltimore, MD, USA (6); Manchester, United Kingdom (7); and Rome, Italy (1), found these mice to be carriers of LCMV, *Toxoplasma gondii*, and pathogenic *Leptospira* spp., respectively, further highlighting the risks that they present to urban centers.

Large urban centers such as New York City (NYC) provide ideal habitats for rodents such as house mice, because the combination of aging, interconnected infrastructure and a dense human population provides ample opportunity for them to thrive (8). Large apartment buildings provide unfettered access to shelter, warmth, and ample food sources, the last often concentrated inside compactor rooms where general waste from the apartments above is consolidated prior to disposal. Mice that have colonized these buildings are shielded from extreme temperatures and have sufficient food to breed year-round (9). The continuous maintenance and interapartment spread of their population, aided by a rapid and prolific breeding cycle, are integral to their commensal lifestyle and a key factor in the high levels of interaction that they have with humans (10).

Here, we report investigation of house mice for the presence of known and novel viruses utilizing a two-tiered discovery approach of broad, unbiased high-throughput sequencing (UHTS) supplemented with targeted molecular screening. We also report surveillance for the presence of serum antibodies to LCMV using an infected-cell enzyme-linked immunosorbent assay (ELISA).

## RESULTS

### Mouse collection.

**FIG 1:**
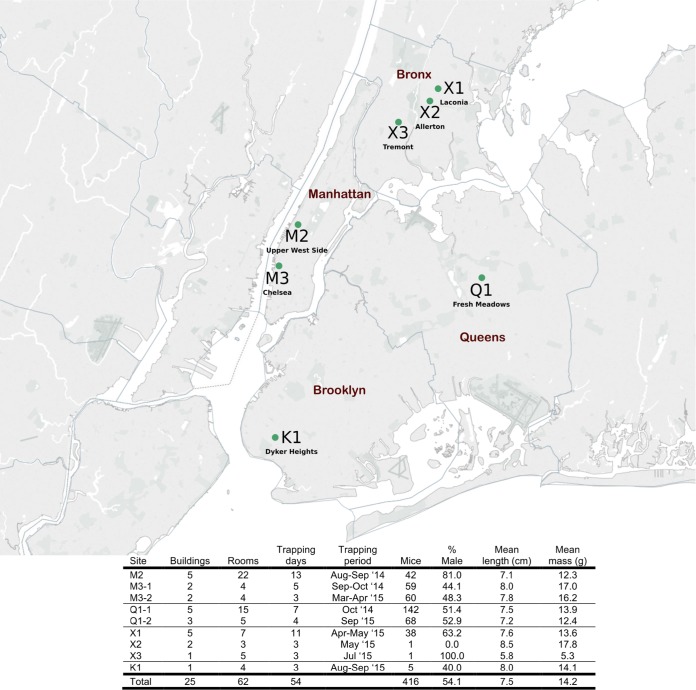
Site locations in New York City and house mouse population summary. Map created with Tableau Software and published with permission of the company.

A total of 416 mice were trapped from seven sites in four boroughs over a 15-month period in NYC (Fig. 1). Mice were caught in or around compactor rooms in the subbasements of residential multifamily housing apartment buildings with the exception of site K1 in Brooklyn, where 5 mice were trapped in food preparation/storage areas of a commercial building, and site X3, where a single mouse was trapped in a private apartment. For sites M3 and Q1, a second site visit occurred 6 and 11 months after the first trapping, respectively (designated site-1 and site-2). Thirty-seven mice from Queens time point 1 (Q1-1) and 1 mouse from time point 2 (Q1-2) were swabbed and bled; however, no organs were collected. In 21 mice, serum volumes were insufficient to complete LCMV ELISAs.

Overall, we trapped more males than females (54% male), especially at site M2 (81%). In total, we collected 194 adults, 70 subadults, and 152 juveniles. Significant differences in the lengths of mice (an indirect measure of age) were observed between sites (one-way analysis of variance [ANOVA], *P* < 0.001), with M3 mice significantly longer than mice in Q1 (*P* = 0.003) and M2 (*P* = 0.001). Furthermore, mice from site M3 were also significantly heavier (independent of length) than mice in Q1 (Δ = 1.08 g; 95% confidence interval [CI], 0.62 to 1.55; *P* < 0.001), X1 (Δ = 1.44 g; 95% CI, 0.70 to 2.18; *P* < 0.001), and K1 (Δ = 2.93 g; 95% CI, 1.12 to 4.75; *P* = 0.002).

### Targeted molecular and serological testing.

Screening for LCMV was carried out using two PCR assays and an ELISA. Neither PCR assay detected active infection in kidney tissues (0/378 samples). ELISAs of sera from 395 mice found no LCMV IgG antibodies.

### Viral discovery.

We assayed pools of 2 to 4 samples representing the fecal pellets of mice trapped individually or in multicatch traps using UHTS. A total of 707,980,718 reads were generated from three lanes of sequencing. Of these reads, 6.2% mapped to the host genome. Using sequence similarity searches on all obtained sequences (assembled sequences and remaining reads, *n* = 138,791,811), 3.0% were annotated as viral sequences, 0.6% were annotated to phages, and a further 31.2% did not return any results. From these data, sequences representing a total of 36 viruses from 18 families and 21 genera were identified. Based upon International Committee on the Taxonomy of Viruses (ICTV) criteria, these included sequences from 6 novel viruses and 3 tentative new genera. Through phylogenetic and sequence similarity analysis, we classified 29 of these viruses as vertebrate associated and 7 as insect associated (Tables 1 and 2). Overall, 2.7% of all reads mapped to the sequences of these 36 viruses. The majority of these reads were sourced from Manhattan mice (M2 and M3-1) and accounted for 68.3% of all viral reads. A total of 3.0% of reads from Bronx and Brooklyn mice mapped to the 36 viruses, compared to 0.6% of Queens (Q1-1) and 7.1% in Manhattan. A single pool from Manhattan dominated the data set with 6.95 million reads (from a total of 9.02 million) mapping to murine-associated porcine bocavirus (MuAPBV). A heat map displaying the mapping of all reads to the sequences of the 36 viruses identified in this study is shown in Fig. S1 in the supplemental material.

10.1128/mBio.01354-17.1 FIG S1  Heat map of UHTS reads mapped against viral sequences found in house mouse fecal pellets. Values represent the log_10_ of specific virus-mapped reads per 10,000,000 reads normalized against total reads per pool. Rows represent individually barcoded pools of fecal pellets from 2 to 4 traps. Download FIG S1, PDF file, 2.3 MB. Copyright © 2018 Williams et al. 2018 Williams et al. This content is distributed under the terms of the Creative Commons Attribution 4.0 International license.

**TABLE 1:** Viruses detected from house mouse fecal samples by UHTS that were not further characterized by PCR^a^

Virus association and name	Family	Genus	Maximum contig (nt)	Most similar viral sequence	Coverage (%)	BLAST identity (%)	E value
Vertebrate associated							
Murine adenovirus 2	*Adenoviridae*	*Mastadenovirus*	20,169	Murine adenovirus 2	99	95	0.0
Mouse papillomavirus 1	*Papillomaviridae*	*Pipapillomavirus*	7,375	Mus musculus papillomavirus 1	100	99	0.0
Murine AAV-1	*Parvoviridae*	*Dependovirus*	4,339	Mouse AAV-1	56	91	0.0
Murine AAV-2	*Parvoviridae*	*Dependovirus*	4,449	Mouse AAV-1	43	75	0.0
Mouse parvovirus 2	*Parvoviridae*	*Protoparvovirus*	4,875	Mouse parvovirus 2	100	99	0.0
Canine parvovirus 2	*Parvoviridae*	*Protoparvovirus*	4,899	Canine parvovirus 2a	97	97	0.0
Murine FaGv-1	*Genomoviridae*	*Gemycircularvirus*	2,129	Mongoose FaGv	53	81	0.0
Murine FaGv-2	*Genomoviridae*	*Gemycircularvirus*	2,418	HCBI9.212 virus	83	77	0.0
Chicken anemia virus	*Anelloviridae*	*Gyrovirus*	2,023	Chicken anemia virus	100	98	0.0
Murine circovirus	*Circoviridae*	*Circovirus*	909	Porcine-circo-like virus 21	89	38^b^	5e−57
TMEV	*Picornaviridae*	*Cardiovirus*	7,986	Sikhote-Alin virus	99	84	0.0
Murine picobirnavirus 1	*Picobirnaviridae*	*Picobirnavirus*	1,011	Picobirnavirus 504	97	75	7e−176
Murine picobirnavirus 2	*Picobirnaviridae*	*Picobirnavirus*	1,544	Human picobirnavirus	77	71	9e−145
Murine picobirnavirus 3	*Picobirnaviridae*	*Picobirnavirus*	1,602	Wolf picobirnavirus	64	71	4e−124
Insect associated							
Fresh Meadows densovirus 1	*Parvoviridae*	*Densovirus*	3,171	Bombyx mori densovirus Zhenjiang	98	34^b^	1e−151
Fresh Meadows densovirus 2	*Parvoviridae*	*Densovirus*	3,189	Bombyx mori densovirus 3	96	35^b^	3e−169
Fresh Meadows densovirus 3	*Parvoviridae*	*Densovirus*	3,027	Bombyx mori densovirus Zhenjiang	99	33^b^	4e−151
Fresh Meadows densovirus 4	*Parvoviridae*	*Densovirus*	3,387	Bombyx mori bidensovirus	91	30^b^	1e−122
Wuchang cockroach virus 3	*Chuviridae*	Unclassified	3,245	Wuchang cockroach virus 3	100	99	0.0
Chelsea phlebo-like virus^c^	*Bunyaviridae*	*Phlebovirus*	4,408	Arumowot virus	13	25^b^	0.031
Bloomfield virus	*Reoviridae*	Unclassified	4,059	Bloomfield virus	98	95	0.0

a Abbreviations: AAV, adeno-associated virus; FaGv, feces-associated gemycircularvirus; TMEV, Theiler’s murine encephalomyelitis virus. Classification is per ICTV except for Wuchang cockroach virus 3 (59). Plant and fungal viruses, including those taxonomically assigned to *Tymovirales*, *Tombusviridae*, *Virgaviridae*, *Partitiviridae*, *Totiviridae*, and *Endornaviridae*, were not included in the analysis. Contigs less than 500 nt were excluded.

b Alignment was performed at the amino acid level (BLASTx).

c Partial genome by Sanger sequencing.

**TABLE 2:** Viruses detected from house mouse fecal samples by UHTS that were further characterized by PCR^a^

Virus name	Abbreviation	Family	Genus	Maximum contig (nt)	Most similar viral sequence	Coverage (%)	BLAST identity (%)	E value
Murine-associated porcine bocavirus^f^	MuAPBV	*Parvoviridae*	*Bocaparvovirus*	4,851	Porcine bocavirus 1	99	90	0.0
Murine bocavirus^f^	MuBV	*Parvoviridae*	*Bocaparvovirus*	5,069	Rat bocavirus	46	49^d^	0.0
Murine chapparvovirus^f^	MuCPV	*Parvoviridae*	*Chapparvovirus^b^*	4,174	Desmodus rotundus parvovirus	84	70	0.0
Mus musculus polyomavirus 3^e^	MmusPyV-3	*Polyomaviridae*	*Betapolyomavirus*	5,091	Rattus norvegicus polyomavirus 2	87	72	0.0
Lactate dehydrogenase -elevating virus	LaDV	*Arteriviridae*	*Porartevirus*	14,052	LaDV	99	88	0.0
Murine hepatitis virus	MHV	*Coronaviridae*	*Betacoronavirus*	31,414	MHV	98	94	0.0
Murine astrovirus 1^f^	MuAst-1	*Astroviridae*	*Mammastrovirus*	6,707	Murine astrovirus- STL 2	100	85	0.0
Murine astrovirus 2^f^	MuAst-2	*Astroviridae*	*Mammastrovirus*	6,197	Rat astrovirus	78	77	0.0
Murine norovirus	MNV	*Caliciviridae*	*Norovirus*	7,382	Murine norovirus 3	100	91	0.0
Murine sapovirus^g^	MuSaV	*Caliciviridae*	*Sapovirus*	7,169	Porcine sapovirus (OH-JJ681)	83	42^d^	0.0
Murine picornavirus^f^	MuPiV	*Picornaviridae*	Unclassified	7,112	Rabovirus A	97	52^d^	0.0
Murine kobuvirus^f^	MuKoV	*Picornaviridae*	*Kobuvirus*	8,099	Mouse kobuvirus	99	78	0.0
Murine feces-associated hepe-like virus^f^	MuFAHLV	Unclassified^c^	Unclassified	7,192	Hubei hepe-like virus	66	37^d^	0.0
Murine rotavirus	MuRotaV	*Reoviridae*	Rotavirus	2,426	Murine rotavirus	99	88	0.0
Murine feces-associated rhabdovirus^f^	MuFARV	*Rhabdoviridae*	Unclassified	11,849	Vesicular stomatitis New Jersey virus	54	38^d^	0.0

a Classification is per ICTV except as noted otherwise. Plant and fungal viruses, including those taxonomically assigned to *Tymovirales*, *Tombusviridae*, *Virgaviridae*, *Partitiviridae*, *Totiviridae*, and *Endornaviridae*, were not included in the analysis. Contigs less than 500 nt were excluded.

b See reference 15.

c Member of a proposed “Hepe-Virga” clade (27).

d Alignment performed at the amino acid level (BLASTx).

e Full genome by Sanger sequencing.

f Full coding regions by Sanger sequencing.

g Near-full coding region by Sanger sequencing.

The majority (23/29) of vertebrate-associated viruses were at least 70% identical to their closest relative at nucleotide level. Of the 29 vertebrate-associated viruses identified in pooled fecal pellets by UHTS, 15 were selected (Table 2) for PCR screening of individual anal swabs (AS) and liver samples to assess overall prevalence, distribution, and diversity. These viruses represented a broad cross section of viral genome types that either belonged to a genus or family that includes viruses known to cause human infection or are unique or novel to house mice. Sanger sequencing was performed on 11 of these viral genomes to confirm UHTS data (Fig. S2). The sequences of the remaining four murine viruses (murine norovirus [MNV], murine rotavirus [MuRotaV], murine hepatitis virus [MHV], and lactate dehydrogenase-elevating virus [LaDV]) were not confirmed with Sanger sequencing because sequences obtained through UHTS were consistent with previous reports (11–14). MNV, MuRotaV, and MHV sequences were obtained directly from UHTS of feces. The near-complete genomic sequence for LaDV, a hepatotropic virus, was obtained from UHTS of liver.

10.1128/mBio.01354-17.2 FIG S2  Annotated genomes of viruses confirmed by Sanger sequencing in house mouse fecal pellets. Numbers represent nucleotide positions; orange arrows indicate open reading frames; green arrows indicate locations of posttranslationally cleaved proteins for members of the viral family *Picornaviridae*. Download FIG S2, PDF file, 1.7 MB. Copyright © 2018 Williams et al. 2018 Williams et al. This content is distributed under the terms of the Creative Commons Attribution 4.0 International license.

### Viral characterization and phylogenetics. (i) Parvoviruses.

A large diversity of parvoviruses was identified from UHTS that included members from the *Parvovirinae* (*n* = 8) and *Densovirinae* (*n* = 4) subfamilies. The arthropod-associated members of the *Densovirinae* subfamily were not explored further.

**FIG 2:**
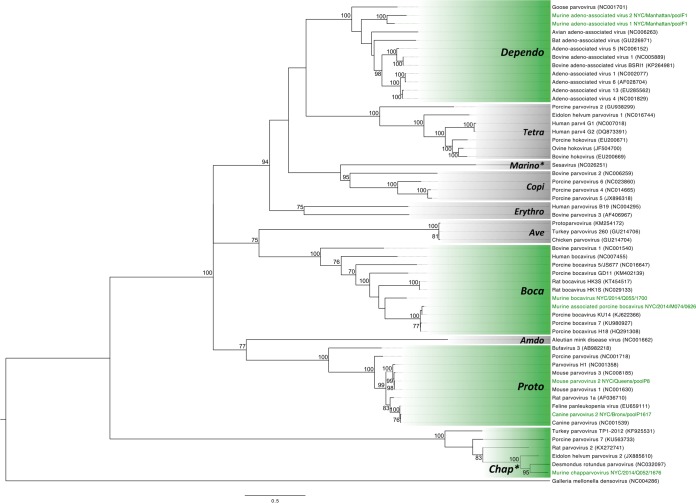
Maximum likelihood phylogenetic tree of the NS1 protein for representative members of the subfamily *Parvovirinae*, including all ICTV-recognized genera as well as the proposed *Chapparvovirus* and *Marinoparvovirus* genera (denoted by asterisks). The scale bar represents units of substitutions per site. Viruses identified in this study and the genera to which they are assigned are labeled in green. All other genera are labeled in a gray box. All bootstrap nodal support values are indicated if >70.

One parvovirus, tentatively named murine chapparvovirus (MuCPV), is a member of a newly proposed genus within the *Parvovirinae* subfamily, *Chapparvovirus* (15). MuCPV is most closely related to Desmodus rotundus parvovirus, sharing 59% amino acid similarity across the nonstructural protein 1 (NS1) and 60% in the capsid. MuCPV was detected in AS samples from all sites, excluding those with the smallest sample numbers (X2, X3, and K1). The prevalence of MuCPV DNA in AS was high relative to other viruses detected in this study; positive results were obtained in 19% of mice from M2, 45% of mice from M3, 44% of mice from Q1, and 13% of mice from X1 (Table 3). MuCPV DNA was detected in liver samples at a higher rate than any other virus, with 34% of all mice being positive (Table 3); 21% of all mice were positive in both the liver and AS (data not shown). The prevalence of MuCPV in liver increased with age: 5% of all juveniles and 62% of adults were positive. NS1 nucleotide sequence identity was high between mice, with all positive samples being >98% identical, irrespective of site. Phylogenetic analysis of the NS1 protein confirmed the close relationship between MuCPV and other members of the *Chapparvovirus* genus (Fig. 2). MuCPV was placed in a well-supported (95% bootstrap nodal support) clade that also included two bat parvoviruses, Eidolon helvum parvovirus 2 and Desmodus rotundus parvovirus, and the more distantly related rat parvovirus 2. According to a recent proposal to the ICTV, species demarcation within the *Parvoviridae* family requires >15% amino acid divergence from other species across the NS1 protein (16). Thus, with 41% divergence, MuCPV represents a tentative new species member of the proposed *Chapparvovirus* genus.

**TABLE 3:** Prevalence of viral nucleic acid sequences detected by PCR from anal swabs and liver tissue

Virus	Sample	% prevalence by PCR^a^												
		Site							Sex		Age			Total
		M2	M3	Q1	X1	X2	X3	K1	M	F	J	SA	A	
MuAPBV	AS		42.9	1					43	57	21	8	72	12.7
	Liver		33.6	0.6					46	54	2	7	90	10.8
MuBV	AS			10					67	33	43	24	33	5
	Liver			0.6					100	0	100	0	0	0.3
MuCPV	AS	19	44.5	44.3	13.2				53	47	24	11	65	38.2
	Liver	23.8	39.5	36	26.3	100			53	47	5	8	87	34.4
MmusPyV-3	AS							40	0	100	50	0	50	0.5
	Liver							20	0	100	100	0	0	0.3
LaDV	AS	4.8	43.7						56	44	15	9	76	13
	Liver	9.5	51.3	0.6					52	48	12	9	79	17.5
MHV	AS		21	4.3					53	47	56	9	35	8.2
	Liver		0.8	0.6					50	50	50	0	50	0.5
MuAst-1	AS	40.5	40.3	21.4	23.7				57	43	42	13	45	28.6
	Liver	21.4	9.2	12.2	5.3				56	44	70	7	23	11.4
MuAst-2	AS	40.5	60.5	33.8				20	55	45	20	15	65	38.7
	Liver	14.3	30.3	16.3				20	55	45	18	31	51	18.8
MNV	AS	26.2		18.1					59	41	22	16	61	11.8
	Liver	4.8		12.8					42	58	42	12	46	6.3
MuSaV	AS	11.9	21.8	7.6					51	49	53	17	30	11.3
	Liver	9.5	16	7					34	66	51	23	26	9.3
MuPiV	AS	16.7		14.8				20	26	74	59	15	26	9.4
	Liver	2.4		1.2					33	67	33	33	33	0.8
MuKoV	AS	23.8	7.6	21.9					66	34	28	18	54	15.6
	Liver	4.8	0.8	1.7					83	17	50	17	33	1.6
MuFAHLV	AS	7.1		1	2.6				67	3	33	17	50	1.4
	Liver								0	0	0	0	0	0
MuRotaV	AS		8.4		5.3	100			15	85	38	31	31	3.1
	Liver		0.8						0	100	0	0	100	0.3
MuFARV	AS			0.5					100	0	0	100	0	0.2
	Liver								0	0	0	0	0	0

a Abbreviations: M, male; F, female; J, juvenile; SA, subadult; A, adult.

Two bocaviruses, the first to be described in *M. musculus*, were also confirmed from fecal pellets. MuAPBV was closely related to porcine bocavirus (91% nucleotide identity and 94% amino acid similarity across the NS1 protein); murine bocavirus (MuBV) was more divergent, sharing only 50% amino acid similarity with rat bocavirus in the NS1 protein gene. These bocaviruses were 55% similar to each other at the amino acid level in the NS1 protein, and each virus was present at different sites (Table 3). PCR of AS demonstrated that MuAPBV was primarily confined to site M3 (43% prevalence) with just two positive mice at Q1 (1% prevalence) (Table 3). The presence of nucleic acid in liver was similarly high at site M3, with 34% of mice positive by PCR (Table 3). Unlike MuAPBV, MuBV was restricted to Q1 (10% prevalence in AS) and was rarely detected in the liver.

Phylogenetic analysis placed both viruses in the *Bocaparvovirus* genus (Fig. 2). MuAPBV clustered closely with porcine bocaviruses, while MuBV was located on a sister branch with respect to MuAPBV. Using the proposed species demarcation cutoff of >15% amino acid distance in the NS1 protein (16), MuAPBV is defined as a member of the *Ungulate bocaparvovirus 4* species, whereas MuBV meets the criteria for a distinct bocaparvovirus.

### (ii) Polyomavirus.

A novel polyomavirus was identified in fecal pellets from a single site in Brooklyn. The complete circular genome of Mus musculus polyomavirus 3 (MmusPyV-3) was 5,091 nucleotides (nt) long and encoded VP1, VP2, and VP3 on one strand as well as the small and large T antigens (LTAg) on the other, with coding regions separated by a presumptive noncoding control region (Fig. S2). Alignment of the LTAg using BLASTn revealed that the virus shared 75% nucleotide identity with Rattus norvegicus polyomavirus 2 (RnorPyV-2) (17). According to ICTV guidelines, this virus meets the definition for classification of a tentative new species as it has (i) a typical polyomavirus genome organization and (ii) an association with *M. musculus*; (iii) the genetic distance to the most closely related species, RnorPyV-2, is >15%; and finally, (iv) the complete genome sequence has been acquired (18). MmusPyV-3 DNA was detected in the AS of 2 mice (one of these mice was also PCR positive in the liver) from site K1 in Brooklyn, resulting in a combined prevalence of 0.5% for all mice (Table 3). Phylogenetic analysis of LTAg placed MmusPyV-3 within the ICTV-recognized *Betapolyomavirus* genus in a well-supported clade (98% bootstrap support) shared with other rodent polyomaviruses, including RnorPyV-2, bank vole polyomavirus, and common vole polyomavirus, as well as the two human-associated viruses, Wu and Ki polyomaviruses (Fig. S3). This finding lends support to the suggestion that the ancestor of this clade may have been found in a rodent (19).

10.1128/mBio.01354-17.3 FIG S3  Maximum likelihood phylogenetic tree of the large T antigen protein of viruses of the family *Polyomaviridae* (sequences obtained from reference 60). The scale bar represents units of substitutions per site. The polyomavirus identified in this study and its associated genus are labeled in green. All other genera are labeled in a gray box. All bootstrap nodal support values are indicated if >70. Download FIG S3, PDF file, 1.5 MB. Copyright © 2018 Williams et al. 2018 Williams et al. This content is distributed under the terms of the Creative Commons Attribution 4.0 International license.

### (iii) Astroviruses.

From each of the four boroughs, astroviral sequences were recovered that shared nucleotide sequence identity with murine and rat astroviruses. Two unique astroviruses, designated murine astrovirus 1 (MuAst-1) and murine astrovirus 2 (MuAst-2), were confirmed following direct PCR that targeted the open reading frame (ORF) 1b-ORF2 junction. The astroviruses were 28% similar to each other across the capsid protein and 52% identical at the nucleotide level within the PCR product. Each assembled sequence contained a typical astrovirus genome structure (Fig. S2). MuAst-1 was most closely related to murine astroviruses found in laboratory mice across North America and shared 88% amino acid similarity over the complete capsid sequence (20). The second astrovirus, MuAst-2, was most closely related (75% amino acid similarity in capsid) to an astrovirus recovered from Norway rats in Hong Kong (astrovirus rat/RS126/HKG/2007) (21). Astrovirus nucleic acid was detected in AS from all sites for both viruses with the exceptions of MuAst-1 in K1 and MuAst-2 in X1/2/3. Prevalence in the remaining sites was high, ranging from 21% to 40% for MuAst-1 and 20% to 60% for MuAst-2 (Table 3). Virus was also detected in liver samples with 11% of all mice being positive for MuAst-1 and 19% for MuAst-2 (Table 3). Fifty-eight mice carried both astroviruses in their AS. Eight mice were PCR positive for each virus in their livers.

Current ICTV-recognized *Mamastrovirus* species are separated by greater than 37.8% amino acid distance in the capsid protein; thus, both astroviruses discovered in this study do not likely constitute novel species (22). Phylogenetic analysis of the capsid protein places MuAst-1 in a clade shared with murine astroviruses (100% bootstrap support). MuAst-2 shares a clade with the two rat astroviruses from Hong Kong (100% bootstrap support) (Fig. S4).

10.1128/mBio.01354-17.4 FIG S4  Maximum likelihood phylogenetic tree of the capsid protein for representative members of the viral family *Astroviridae*. The scale bar represents units of substitutions per site. Genera are labeled with a black circle, and recognized species are marked in gray boxes (MAstV, mammalian astrovirus; AAstV, avian astrovirus). Viruses identified in this study and the unclassified clades to which they are assigned are labeled in green. All bootstrap nodal support values are indicated if >70. Download FIG S4, PDF file, 1.4 MB. Copyright © 2018 Williams et al. 2018 Williams et al. This content is distributed under the terms of the Creative Commons Attribution 4.0 International license.

### (iv) Sapovirus.

A novel sapovirus (murine sapovirus [MuSaV]) was discovered in fecal pellets from Manhattan (M2 and M3) and Queens (Q1) with 42% amino acid similarity to porcine sapovirus across the complete polyprotein and 54% amino acid similarity across the major capsid protein (VP1) to a partially sequenced rodent sapovirus identified in brown rats from NYC (sapovirus 1 rodent/Manhattan/Ro-SaV1) (23). This is the first sapovirus reported in house mice. The similarity in the VP1 protein (54%) is less than the proposed 57% cutoff used to define a new genogroup (24); therefore, MuSaV may warrant the creation of a 16th genogroup within the *Sapovirus* genus. Phylogenetic analysis of VP1 protein sequence for all sapovirus genogroups supports the creation of a tentative new genogroup, as MuSaV is found on a deeply rooted branch with Ro-SaV1 as its closest neighbor (sole member of genogroup XV) (100% bootstrap support) (Fig. S5). The near-complete coding sequence for MuSaV demonstrated a genome structure consistent with other sapoviruses, including two ORFs where ORF2 (VP2) overlaps ORF1 in a −1 frameshift (Fig. S2). Of 13 conserved amino acid motifs previously identified in all sapovirus species, 9 were maintained while the remaining 4 had single-amino-acid changes (PL[N/D]CD→**V**L[N/D]CD) in NS3, XDEYXX→XD**D**YXX in NS5, and WKGL→W**R**GL and GLPSG→G**I**PSG in NS7) (24). The putative NS7-VP1 cleavage site was YVME/G based on amino acid alignments of ORF1 polyprotein with reference sequences. MuSaV was not detected in mice from the Bronx or Brooklyn sites; however, the remaining three sites in Manhattan and Queens were positive with prevalences ranging between 8% (Q1) and 22% (M3) (Table 3). Juvenile mice were the most frequently positive, comprising 53% of all positive AS samples. MoSaV RNA was detected in 9% of all mouse livers (Table 3).

10.1128/mBio.01354-17.5 FIG S5  Maximum likelihood phylogenetic tree of VP1 for members of the viral family *Caliciviridae*. The scale bar represents units of substitutions per site. Genera are labeled with a black circle. Representative viruses from currently recognized genogroups for the *Sapovirus* and *Norovirus* genera are shaded in gray boxes. Viruses identified in this study and the associated genogroups are labeled in green. All bootstrap nodal support values are indicated if >70. Download FIG S5, PDF file, 1.4 MB. Copyright © 2018 Williams et al. 2018 Williams et al. This content is distributed under the terms of the Creative Commons Attribution 4.0 International license.

### (v) Picornaviruses.

**FIG 3:**
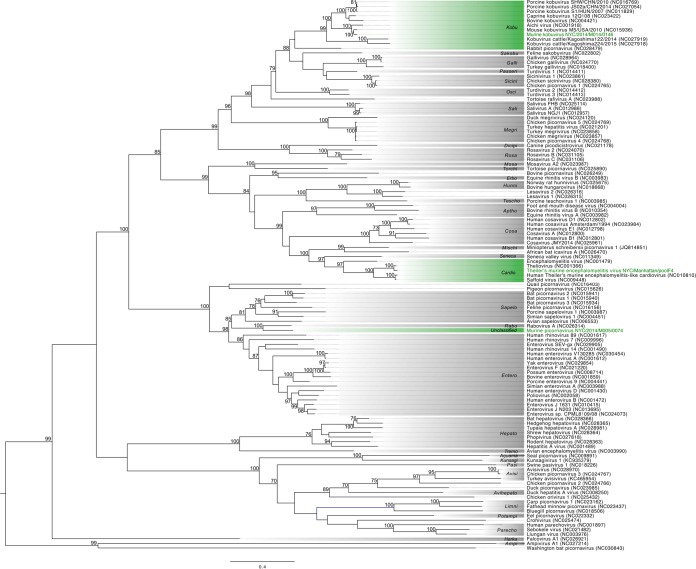
Maximum likelihood phylogenetic tree of the 3D polymerase protein of all recognized *Picornaviridae* genera. The scale bar represents units of substitutions per site. Viruses identified in this study and the genera to which they are assigned are labeled in green. All other genera are labeled in a gray box. All bootstrap nodal support values are indicated if >70.

Murine picornavirus (MuPiV)—identified in fecal pellets in Manhattan, Queens, and Brooklyn—displayed 52% amino acid similarity across the polyprotein to rabovirus A, a picornavirus detected in Norway rats that belongs to the newly created *Rabovirus* genus (25). MuPiV is closely related to mouse sapelovirus M-58/USA, a partially sequenced virus detected in the feces of a house mouse from Virginia, USA (26). There was 77% nucleotide identity and 88% amino acid identity between the two viruses within the short partial VP4-2 sequence that is publicly available. Phylogenetic analysis of the MuPiV 3D polymerase indicates that it shares a common ancestor with rabovirus A (100% bootstrap support), nested between the *Sapelovirus* and *Enterovirus* genera (Fig. 3). MuPiV may represent a tentative new *Picornaviridae* genus with amino acid similarity across the polyprotein (52%) less than the 58% cutoff defined by the ICTV (22).

MuPiV displayed marked heterogeneity between sample sites with three clear genotypes, sharing between 62% and 89% nucleotide identity in the VP1 region based on available sequence from the screen PCR (270 nt). MuPiV was detected in 9% of mouse AS with the majority of viruses found in female (74% of all detections) and juvenile (59%) mice (Table 3). No evidence of MuPiV was found in the Bronx mice (X1, X2, and X3) or at the M3 site in Manhattan; however, all remaining sites were positive with prevalences ranging between 15% (Q1) and 20% (K1). Viral nucleic acid was detected in less than 1% (3/378) of mouse livers (Table 3).

A second picornavirus, murine kobuvirus (MuKoV), was detected in feces from both Manhattan sites (M2 and M3) and Queens (Q1). At the amino acid level, MuKoV is 83% similar over the full polyprotein, 93% similar in the 3D polymerase, and 71% similar in VP1 to mouse kobuvirus M-5/USA/2010, a virus identified from a canyon mouse (*Peromyscus crinitus*) in California, USA (26). The genome structure is consistent with that of other kobuviruses (Fig. S2), and based on the amino acid similarity of the polyprotein, P1, 2C, and 3CD, as well as phylogenetic placement, MuKoV is a member of the *Aichivirus A* species within the *Kobuvirus* genus (Fig. 3). PCR screening of AS samples revealed widespread prevalence of MuKoV across three sites from two boroughs (M2, 24%; M3, 8%; and Q1, 22%) (Table 3). MuKoV cDNA was detected in 6/378 liver samples (Table 3).

### (vi) Hepe-like virus.

A highly divergent virus most closely related to hepeviruses and other unclassified members of a newly proposed *Hepe-Virga* clade (27) was identified in three NYC boroughs. Murine feces-associated hepe-like virus (MuFAHLV) shares 37% amino acid similarity to Hubei hepe-like virus 3 (HHLV-3) across the near-complete replicase and 29% similarity to swine hepatitis E virus. Conserved domain searches within the replicase revealed a viral methyltransferase, viral helicase (superfamily 1), and RNA-dependent polymerase (RdRp; superfamily 2) domain. The capsid is contained in a 483-amino-acid (aa) open reading frame nested within the replicase in a +1 frameshift and shared 35% amino acid similarity with HHLV-3 (Fig. S2). A 228-aa hypothetical protein is encoded in a −1 frameshift relative to the replicase gene with a single-nucleotide overlap. The hypothetical protein does not contain any conserved domains and does not share any recognizable homology to other viruses. MuFAHLV sequences were detected in fecal pellets from M2 (7% prevalence), Q1 (1%), and X1 (3%) (Table 3). No sequences were detected in liver samples. Sequencing of the screening PCR product that targeted a 311-nt conserved region within the RdRp domain identified two major genotypes that were between 75.4% and 76.5% identical. Both genotypes were found in M2, whereas only a single related genotype was detected at Q1 and X1. Phylogenetic analysis using concatenated conserved regions of the helicase and polymerase domains places MuFAHLV into an unclassified clade shared with HHLV-3 (Fig. S6). These two viruses share a common ancestor with members of the *Hepeviridae* that include hepatitis E virus, avian hepatitis E virus, and the recently described cutthroat trout virus (28).

10.1128/mBio.01354-17.6 FIG S6  Maximum likelihood phylogenetic tree of concatenated helicase/polymerase proteins for representative members of the Hepe-Virga clade. The scale bar represents units of substitutions per site. The hepe-like virus identified in this study and the clade to which it is assigned are labeled in green. All other recognized viral families are labeled in a gray box. All bootstrap nodal support values are indicated if >70. Download FIG S6, PDF file, 1.4 MB. Copyright © 2018 Williams et al. 2018 Williams et al. This content is distributed under the terms of the Creative Commons Attribution 4.0 International license.

### (vii) Rhabdovirus.

**FIG 4:**
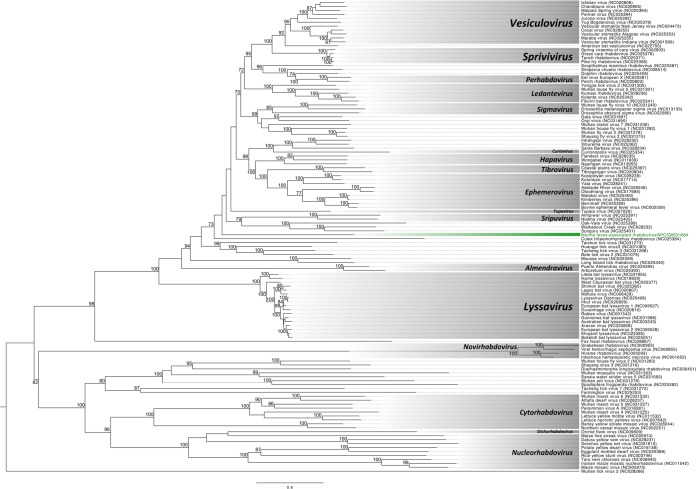
Maximum likelihood phylogenetic tree of the complete L protein for rhabdoviruses. The scale bar represents units of substitutions per site. The rhabdovirus identified in this study and the unclassified clade to which it is assigned are labeled in green. All other recognized genera are labeled in a gray box. All bootstrap nodal support values are indicated if >70.

A highly divergent rhabdovirus, provisionally named murine feces-associated rhabdovirus (MuFARV), was identified in a single mouse trapped in Queens (Q1). The obtained sequence shared a similar genome architecture with members of the *Rhabdoviridae*, with five nonoverlapping ORFs organized as 3′-nucleoprotein-phosphoprotein-matrix-glycoprotein-polymerase-5′ separated by four intergenic regions (66 nt, 94 nt, 68 nt, and 63 nt, respectively), with each region containing transcription termination (CATGAAAAAAA) and initiation (TAAC[A]ARR) sites (Fig. S2). MuFARV is most similar to vesicular stomatitis New Jersey virus across the polymerase (38%), glycoprotein (22%), and nucleoprotein (27%); the putative matrix and phosphoproteins were dissimilar to any known sequence by unrestricted BLASTp similarity searches. We were unable to place MuFARV into any genus currently recognized by ICTV through phylogenetic analysis of the polymerase (L) protein (Fig. 4). MuFARV is located on a monophyletic branch that is rooted in a posterior position relative to the *Vesiculo-*, *Sprivi-*, *Perhabdo-*, *Ledante-*, *Sigma-*, *Curio-*, *Hapa-*, *Tibro-*, *Ephemero-*, *Tupa*-, and *Sripuvirus* genera. PCR screening of all available livers and AS provided no further evidence of this virus in any other mouse, aside from the fecal pellet and AS sourced from the single Q1 mouse.

### Viral persistence at trapping sites.

Virus PCR screening results were compared for two sites that were resampled after 6 (M3) and 11 (Q1) months (Fig. S7). The two astroviruses showed disparate patterns of persistence whereby the prevalence of MuAst-1 increased and that of MuAst-2 decreased across both sites. Of the 14 viruses detected at least once at both sites, only MuAPBV, MHV, MuFAHLV, and MuFARV either emerged or disappeared from detection at a particular site. The only virus to show a significant association with a particular collection time point was MHV at site Q1, where prevalence rose from 0.0% to 13.2% over an 11-month period (odds ratio [OR], 42.7; 95% confidence interval [CI], 4.2 to 5,554.8; *P* = 0.00003). For the remaining 10 viruses, prevalence fluctuated by a maximum of 20%, suggesting that these viruses have established stable infection cycles.

10.1128/mBio.01354-17.7 FIG S7  Change in prevalence of fecal virus detections by PCR between two time points at two sites in NYC. Download FIG S7, PDF file, 2.1 MB. Copyright © 2018 Williams et al. 2018 Williams et al. This content is distributed under the terms of the Creative Commons Attribution 4.0 International license.

### Viral distribution and coinfection burden.

Statistical analyses were used to determine whether site of collection, sex, weight, or length of the mouse was associated with an increased risk of finding a particular virus detected in AS samples. Length and sex were not significantly associated with an increased risk for any virus (data not shown). After controlling for length, sex, and site, a higher weight was associated with a higher likelihood of detection for MuCPV (odds ratio = 1.18; 95% CI, 1.06 to 1.32; *P* = 0.002) or MuAst-2 (odds ratio = 1.18; 95% CI, 1.06 to 1.32; *P* = 0.002). We found no other associations between weight and the presence of other viruses. The prevalence of 12 viruses showed a significant association with a certain site or sites via pairwise comparisons (Table S1). Three viruses (MuAPBV, LaDV, and MHV) were significantly associated with a single site, M3, compared with M2, Q1, and X1.

10.1128/mBio.01354-17.8 TABLE S1  Pairwise association between virus prevalence and site. Download TABLE S1, PDF file, 0.05 MB. Copyright © 2018 Williams et al. 2018 Williams et al. This content is distributed under the terms of the Creative Commons Attribution 4.0 International license.

To determine the impact of location on viral richness, we calculated the total number of viruses found in individual mice by site (Table 4). Eighty-two percent of mice were positive for at least one virus in feces; 61% were positive for at least one virus in liver. There were striking differences between the overall virus coinfection levels from trapping sites in NYC (Table 5). Mice from M3 (Chelsea) carried the most viruses in AS (2.9 viruses/mouse) and liver tissue (1.8 viruses/mouse). Conversely, mice from Bronx site X1 (Eastchester) carried the fewest viruses in each sample type (0.4 and 0.3 virus/mouse, in feces and liver, respectively). Virus richnesses were compared between sites after adjusting for sex, weight, and length using a Poisson regression model. M3 was found to have significantly more viruses per mouse in AS than M2, Q1, and X1 while site X1 had significantly fewer viruses than M2, M3, and Q1 (Table 6).

**TABLE 4:** Viral coinfection burden as detected by PCR

No. of viruses	No. (%) of samples	
	AS	Liver
0	76 (18.2)	162 (38.9)
1	120 (28.9)	138 (33.2)
2	80 (19.2)	74 (17.8)
3	72 (17.3)	31 (7.4)
4	32 (7.7)	10 (2.4)
5	18 (4.3)	1 (0.2)
6	17 (4.1)	0 (0)
7	1 (0.2)	0 (0)

**TABLE 5:** Average number of viral coinfections found in house mouse populations from NYC trapping sites

Site	Avg no. of coinfections in sample type	
	AS	Liver
K1	0.8	0.4
M2	1.9	0.9
M3	2.9	1.8
Q1	1.8	0.7
X1	0.4	0.3
X2	1.0	1.0
X3	0.0	0.0

**TABLE 6:** Pairwise comparison of viral coinfection load between sites

Site pairwise comparison	Fold change	95% confidence interval		*P* value
		Lower limit	Upper limit	
M3 vs M2	1.4	1.1	1.8	5.6 × 10^−3^^a^
M3 vs Q1	1.5	1.3	1.2	6.9 × 10^−7^^a^
M3 vs X1	6	3.7	9.8	9.8 × 10^−13^^a^
M3 vs K1	3.2	1.2	8.5	2.3 × 10^−2^
X1 vs M2	0.2	0.1	0.4	9.7 × 10^−8^^a^
X1 vs Q1	0.2	0.2	0.4	1.9 × 10^−8^^a^
X1 vs K1	0.5	0.2	1.6	0.3
Q1 vs M2	1	0.8	1.2	0.8
Q1 vs K1	2.1	0.8	5.7	0.1
K1 vs M2	0.5	0.2	1.2	0.1

a Statistical significance controlling familywise error rate at the 0.05 level. Sites X2 and X3 were excluded due to an insufficient number of mice for analysis.

The sex, weight, and length of the mouse were also compared with viral coinfection burden within the gut virome. Mouse sex and length were not significantly associated with a difference in total viral burden (as measured by fold change between groups [data not shown]); however, independent of mouse length, weight (as measured in grams) was positively associated with the number of viruses detected in the AS (1.048-fold; 95% CI, 1.014 to 1.083; *P* = 0.005).

We also assessed whether patterns of virus coinfection varied across sites. Overall, we could not reject the null hypothesis that the pattern of viral coinfection was random (*P* = 0.470). However, some viruses had positive or negative associations with one another. LaDV was likely to cooccur with MuAPBV (|Z-score|, 4.34; adjusted *P* < 0.001) but unlikely to cooccur with MNV (|Z-score|, 3.68; adjusted *P* = 0.001) or MuPiV (|Z-score|, 3.67; adjusted *P* = 0.001). MNV was also unlikely to cooccur with MuAPBV (|Z-score|, 3.77; adjusted *P* < 0.001).

## DISCUSSION

Unbiased high-throughput sequence analysis of NYC house mice yielded a diverse collection of novel and known viruses. While 7 of these viruses are likely insect associated, 19 of the remaining 29 vertebrate-associated viruses are either newly described or not previously associated with house mice. The discovery of a diverse array of viruses in wild urban mice was not unexpected. Recent virome studies of rodents have uncovered a broad diversity of previously uncharacterized viruses (26, 29); in earlier studies of NYC Norway rats, we found 18 novel viral sequences (23).

Although we detected no sequences of human viruses, we found sequences in feces that had a high similarity to canine parvovirus, chicken anemia virus, and porcine bocavirus. Canine parvovirus and chicken anemia virus sequences may only represent contaminants in food that mice were consuming. Indeed, we cannot comment on host relationships for any virus discovered exclusively in fecal material (MuFAHLV and MuFARV), as they may also represent food contaminants. However, follow-up PCR screening of tissues revealed that murine-associated porcine bocavirus (MuAPBV) was also present in liver, indicating that this virus is capable of infecting mice. This finding is consistent with the recent report of a bocavirus infection in brown rats in China (30).

MuAPBV represents a tentative new strain of the *Ungulate bocavirus 4* species and is most closely related to PBov-KU14, a virus detected in serum from pigs with respiratory illnesses in South Korea (31). To date, MuAPBV is the only nonswine member of the *Ungulate bocavirus 4* species. The highest prevalence for MuAPBV was in Chelsea, a site that is located close to the Meatpacking District, a neighborhood that contained a high concentration of meat processing facilities as recently as 2003. Whether porcine bocavirus causes disease in pigs is controversial (reviewed in reference 32). This virus has been detected in the neurons of a piglet with encephalomyelitis (33) and has been experimentally shown to interfere with a key interferon signaling pathway (34); however, the high rate of viral coinfections in pigs has made it difficult to confirm a direct association with disease (32). Human bocaviruses have also been linked to disease, including pneumonia and other respiratory infections (35, 36).

Two previously uncharacterized viruses reported here may provide insights into the age distribution of parvoviruses and sapoviruses. The prevalence of murine chapparvovirus (MuCPV) was higher in the livers of adult (62%) than juvenile (5%) mice. In humans, parvovirus B19 infection in children is associated with respiratory disease and rash (fifth disease). In adults, parvovirus B19 has been found to persist in liver (37) and may be associated with acute liver damage (38). In contrast, the murine sapovirus (MuSaV) was detected more frequently in juvenile mice. Human sapoviruses are associated with acute gastroenteritis and infect people of all ages (39). Studies of porcine sapoviruses suggest that genogroup-specific immunity emerges early in life, preventing reinfection (40).

We found two astroviruses that shared 28% amino acid similarity in the capsid protein. In some instances, the two viruses were present within the same mouse. Several other astroviruses had been described in house mice, including M-52/USA/2008 from wild mice in Virginia (26) and MuAst STL1, -2, -3, and -4 from laboratory mice in North America and Japan (20, 41). The detection of MuAst-1 in wild NYC mice and its phylogenetic placement in a clade shared with laboratory mouse astroviruses indicate that these viruses share a common ancestor. The second astrovirus detected in this study, MuAst-2, was instead more closely related to rat astroviruses and formed a separate monophyletic clade. Together, these data suggest that the diversity of astroviruses in house mice may be underappreciated.

Mouse weight (but not length) was positively associated with viral diversity. Chelsea mice, heavier than mice from other sites, harbored the most diverse viromes. This diversity did not appear to adversely impact mouse longevity. The longest mice in this study were trapped in Chelsea; length is commonly used as a correlate of age.

There was no evidence of LCMV infection using molecular methods or serology. LCMV, an uncommon cause of aseptic meningitis in immunocompetent individuals and of life-threatening infections in those who are immunocompromised (42), is the only zoonotic virus currently associated with house mice (2). Aside from an outbreak of 57 cases in 1973 to 1974 that was linked to hamsters (43), recently reported cases of LCMV in New York State are rare but include an individual infection in NYC in 2009 (44) and two cases in children in 2002 and a third in 2009 in Syracuse, NY (45).

House mice are unlike most urban rodents in that they primarily nest within or on the immediate exterior perimeters of built structures, where they intimately coexist with the human population (10). Accordingly, we undertook this project to understand the risks that they may (or may not) pose for human disease in urban centers. While we found no viruses that were closely related to human viruses, we did find evidence of infection with a virus that may have moved from pigs to mice, providing an example of cross-species transmission.

## MATERIALS AND METHODS

### Mouse collection and processing.

Mice were predominantly trapped in the subbasement of medium-sized (5- to 6-level) residential buildings in the four most populated boroughs of NYC: Manhattan, Queens, Brooklyn, and the Bronx (46). A combination of single-catch (SFA folding trap; Sherman, Tallahassee, FL, USA) and multiple-catch (Pro-Ketch; Kness, Albia, IA, USA; and Tin Cat; Victor, Lititz, PA) traps were baited, secured in the open position, and left out on the first night to allow mice access to the trap without being caught and to thus facilitate and expedite colony familiarity and help maximize successful trapping events. On the second day, traps were wiped clean of all fecal matter and urine, rebaited, and set. During inspection on the third day, any traps containing mice were collected, recorded, and transferred to the laboratory.

Following euthanasia by exposure to a lethal dose of CO_2_ per the American Veterinary Medical Association guidelines, mice were weighed and measured from the tip of the nose to the base of the tail. Mouse age was stratified into three categories (juvenile, subadult, and adult) using the length of the mouse (<72 mm, 72 to 77 mm, and >77 mm, respectively) (47). Immediately following euthanasia and recording of mouse weight and length, blood was collected by cardiac puncture and transferred into Microtainer blood collection SST tubes (Becton, Dickinson, Lincoln Park, NJ) containing serum clot activator. Mice were sampled by swabbing of the rectum and tissue harvesting, and fecal pellets were removed from traps. When multiple-catch traps caught more than one mouse, fecal pellets retrieved from the trap were labeled as “pooled” and therefore may represent the fecal material from one or more mice found within that trap. All samples were snap-frozen on dry ice and stored at −80°C. Procedures described here were approved by the Columbia University Institutional Animal Care and Use Committee (protocol number AC-AAAE8351/AC-AAAE8450).

### High-throughput sequencing.

Fecal pellets from a single trap (representing one or multiple mice) were emulsified in phosphate-buffered saline, passed through an 0.45-μm filter (Merck Millipore, Cork, Ireland), and treated with RNase A and Benzonase to digest free nucleic acids prior to lysis and purification on the NucliSens easyMAG automated platform (bioMérieux, Boxtel, The Netherlands). Total nucleic acid was reverse transcribed and RNase H treated prior to pooling with a second fecal pellet sample sourced from another trap and second-strand synthesis. Using the Focused-Ultrasonicator E210 (Covaris, Woburn, MA), double-stranded cDNA was mechanically sheared to an average length of 200 nt, purified, and pooled once more (i.e., total of 4 samples) if low concentrations (<1.5 ng/μl) were observed. Libraries were prepared for sequencing on the HiSeq 2500 system (Illumina, San Diego, CA) using the Hyper Prep kit (Kapa Biosystems, Boston, MA), and sequenced on three lanes: one for Manhattan mice (sites M2 and M3, time point 1), one for Queens mice (site Q1, time point 1), and one for Bronx (sites X1, X2, and X3) and Brooklyn (K1) mice.

Resulting Q30-filtered FastQ files were used to generate quality control reports using PRINSEQ software (v0.20.2) (48) and were further filtered and trimmed. Host sequences were removed by mapping the filtered reads against mouse reference genomes using Bowtie 2 (v2.0.6, http://bowtie-bio.sourceforge.net) (49). The remaining reads were *de novo* assembled using MIRA (4.0) Assembler (50), and contigs and unique singletons were subjected to a similarity search using MegaBLAST against the GenBank nonredundant nucleotide database. Sequences were screened by BLASTx against the viral GenBank protein database if they showed little or no similarity at the nucleotide level. Viral sequences from BLASTx analysis were subjected to another round of BLASTx similarity search against the entire GenBank protein database to correct for biased E values and taxonomic misassignments. All 100-bp, single-end reads were mapped with Bowtie 2 (v2.1.0) against the available genomic sequences of the 36 identified viruses (Tables 1 and 2). The bam files were parsed using BEDTools (v2.26.0), and perl scripts were employed to obtain the viral abundance. Virus-mapped reads were normalized relative to total reads for each pool and compared using a heat map prepared in Microsoft Excel.

### Nucleic acid extraction and PCR.

Nucleic acid was extracted from liver and kidney tissues using the AllPrep DNA/RNA minikit (Qiagen, Valencia, CA) and from AS using the easyMAG automated platform (bioMérieux). Nucleic acid concentration and purity were determined on the NanoDrop 1000 spectrophotometer (Thermo Scientific, Wilmington, DE), and ≤5 µg was used for subsequent cDNA synthesis and PCR testing.

Fifteen viruses identified from analysis of UHTS data were selected for direct PCR screening of AS and liver cDNA. PCR primers for all 15 viruses were manually designed from the obtained UHTS data using Geneious 10.1.2 (51) (see Table S2 in the supplemental material). Direct PCR for LCMV was performed on kidney cDNA using a species-specific assay targeting the S segment (designed for this study) and an arenavirus consensus assay to target the L segment (52). All extracted nucleic acid was tested for inhibitors by performing PCR for host targets glyceraldehyde-3-phosphate dehydrogenase (cDNA) (53) and *M. musculus* mitochondrial d-loop (DNA) (54). Primer sequences, cycling conditions, and gene targets for all PCR assays are detailed in Table S2. All positive PCR results, excluding inhibitor-check PCRs, were confirmed by Sanger sequencing.

10.1128/mBio.01354-17.9 TABLE S2  PCR primers and cycling conditions. Download TABLE S2, PDF file, 0.1 MB. Copyright © 2018 Williams et al. 2018 Williams et al. This content is distributed under the terms of the Creative Commons Attribution 4.0 International license.

### LCMV ELISA.

An IgG assay was used to detect the presence of anti-LCMV antibodies in mouse serum. LCMV antigen (gamma-irradiated lysate obtained from LCMV-infected Vero E6 cells) was applied as a coating to the solid phase of a microtiter plate. Diluted mouse sera (1:160) were added to the first row of the microtiter plate and serially diluted until a 4-fold dilution series was created. The mouse sera were allowed to bind to the antigen, and after washing, an anti-mouse IgG (Thermo Pierce, Waltham, MA) conjugated to horseradish peroxidase was applied and allowed to bind. Plates were washed, the substrate 2′,2-azinobis(3-ethylbenzothiazoline-6-sulfonic acid)-diammonium salt was added, and the plates were read using a preprogrammed BioTek PowerWave 340 reader with Gen 5 software (BioTek, Winooski, VT) at the dual wavelengths of 410 and 490 nm after 30-min incubation at 37C. A mock-infected antigen was used to adjust for background that might be present in the initial substrate. The optical density (OD) values of the normal antigen wells were subtracted from those of the positive antigen to give a net positive adjusted OD value. A positive IgG result was recorded when a sample exhibited a titer of ≥1:400 and a sum OD (calculated by the addition of all four of the sample dilutions) of ≥0.95.

### Phylogenetics.

Viral sequences used for phylogenetic analyses were either confirmed by PCR or directly sourced from UHTS data. Nucleotide sequences were translated and aligned with representative sequences using ClustalW within Geneious 10.1.2 (51) and manually adjusted as required. Alignments were exported into MEGA7 (55) where the model selection algorithm was used to select the best-fitting model for each alignment. Maximum likelihood trees were assembled using a discrete gamma distribution (+G), sometimes coupled with invariant sites (+I) and/or using the nondefault amino acid frequencies of the model (+F) with 500 bootstraps. Newick trees were exported to FigTree (http://tree.bio.ed.ac.uk/software/figtree/) for annotation. Final trees display bootstrap support values when they are above 70%.

### Statistical analyses.

Data were analyzed using Matlab and Statistics Toolbox release 2013a (The MathWorks, Natick, MA). Multiple comparisons and *post hoc* analyses were corrected using Hochberg’s step-up procedure (56) controlling the familywise error rate at a level of α = 0.05.

Demographic measures, including length, weight, and sex, were compared between sites. One-way analysis of variance (ANOVA) was used to determine whether the length of the mice in any site was significantly different from the length of those in any other site. *Post hoc* analysis was conducted to find significant pairwise comparisons. A linear regression model was fitted using weight as the dependent variable and multicategorical site variable as the independent variable, adjusting for length. Finally, the distribution of sex was also compared between sites using a chi-square test with *post hoc* analysis.

For each virus detected, we tested the association between its presence and site or demographic variables by fitting a logistic regression model using the binary virus presence (versus absence) status as the dependent variable and using site, length, weight, and sex as independent variables. Because not all viruses were not found at all sites, we applied Firth logistic regression (57) to deal with the quasicomplete separation phenomenon. Adjustments were made for multiple comparisons (16 viruses and 10 pairwise site comparisons).

We also tested the association between virus richness (i.e., the number of different viruses) and site or demographic variables. The count of viruses was fitted into a Poisson regression model as the dependent variable, and site, length, weight, and sex were used as independent variables. The familywise error rate was controlled at the 0.05 level for the 10 pairwise site comparisons.

Patterns of viral cooccurrence were examined using the Fortran program PAIRS (v1.1) (58) with a fixed-fixed randomization algorithm. Controlling the false discovery rate at an 0.01 level using the Benjamini-Yekutieli procedure, attractive or repulsive relationships between individual pairs of viruses were investigated and considered significantly nonrandom when the absolute Z-score was greater than 3.5 with an adjusted *P* value of <0.01.

### Accession number(s).

The GenBank accession numbers for viruses sequenced in this study are MF175072 to MF175082 (Sanger-sequenced viruses, *n* = 11) and MF416371 to MF416405 (remaining viruses with sequence identified from UHTS data, *n* = 25). The nucleotide sequences for all PCR screening amplicons (*n* = 1247) can be found in Data Set S1.

10.1128/mBio.01354-17.10 DATA SET S1  Sequence data for PCR screening assays. Download DATA SET S1, XLSX file, 0.1 MB. Copyright © 2018 Williams et al. 2018 Williams et al. This content is distributed under the terms of the Creative Commons Attribution 4.0 International license.
