# Supplementary material for: Viral Diversity of House Mice in New York City
Source: mBio. 2018 Apr 17;9(2):e01354-17. doi: 10.1128/mBio.01354-17 (PMC5904411; doi:10.1128/mBio.01354-17)

Fig. S1. Heatmap of UHTS reads mapped against viral sequences found in house mouse fecal pellets. Values represent the $\log_{10}$ of specific virus-mapped reads per 10,000,000 reads normalized against total reads per pool. Rows represent individually barcoded pools of fecal pellets from 2-4 traps.

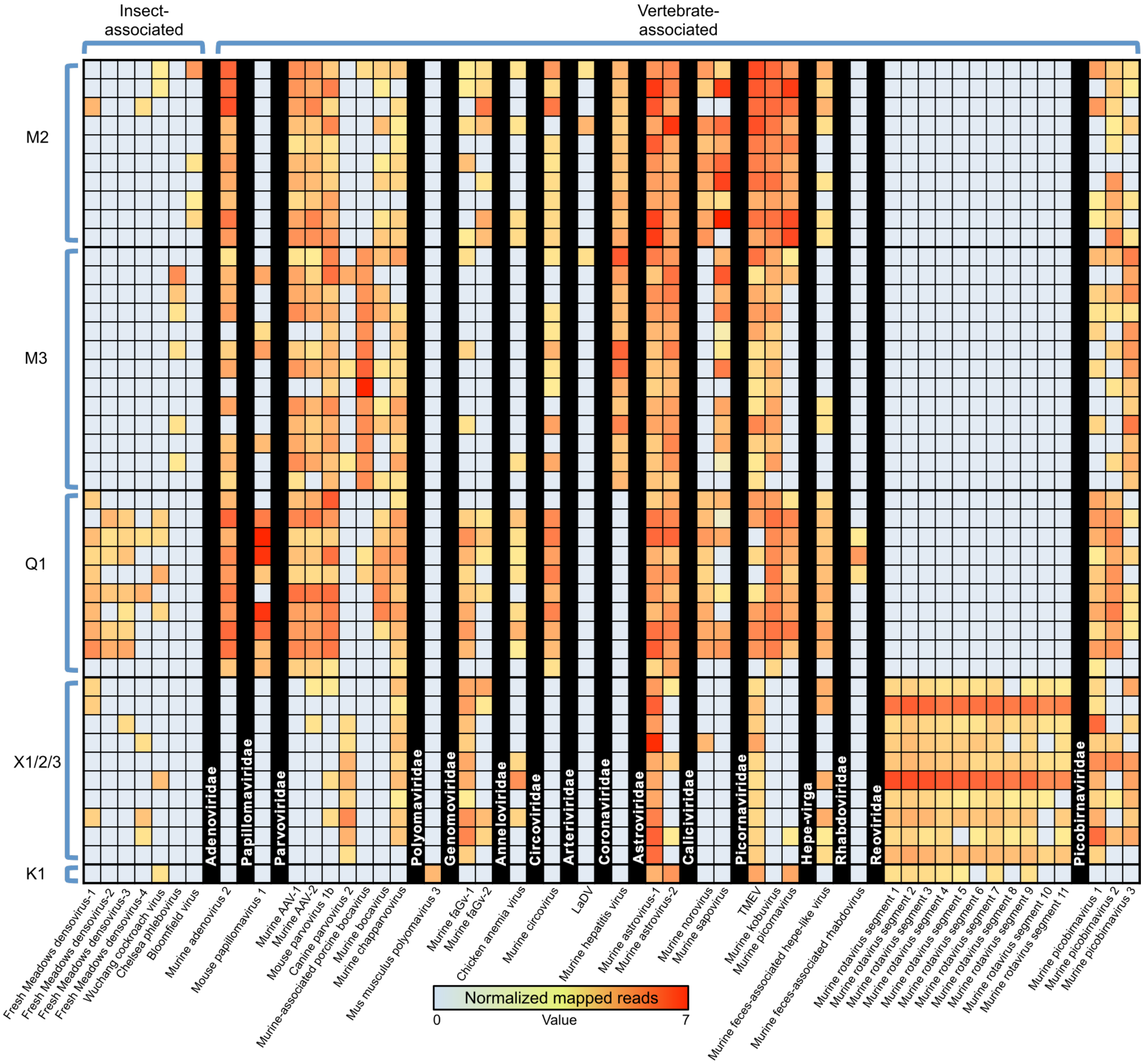

Supplement: FIG S1 [file mbo006173635sf1.pdf]
