# Supplementary material for: Viral Diversity of House Mice in New York City
Source: mBio. 2018 Apr 17;9(2):e01354-17. doi: 10.1128/mBio.01354-17 (PMC5904411; doi:10.1128/mBio.01354-17)

Fig. S2. Annotated genomes of viruses confirmed by Sanger sequencing in house mouse fecal pellets. Numbers represent nucleotide positions; orange arrows indicate open reading frames; green arrows indicate locations of post-translationally cleaved proteins for members of the viral family *Picornaviridae*.

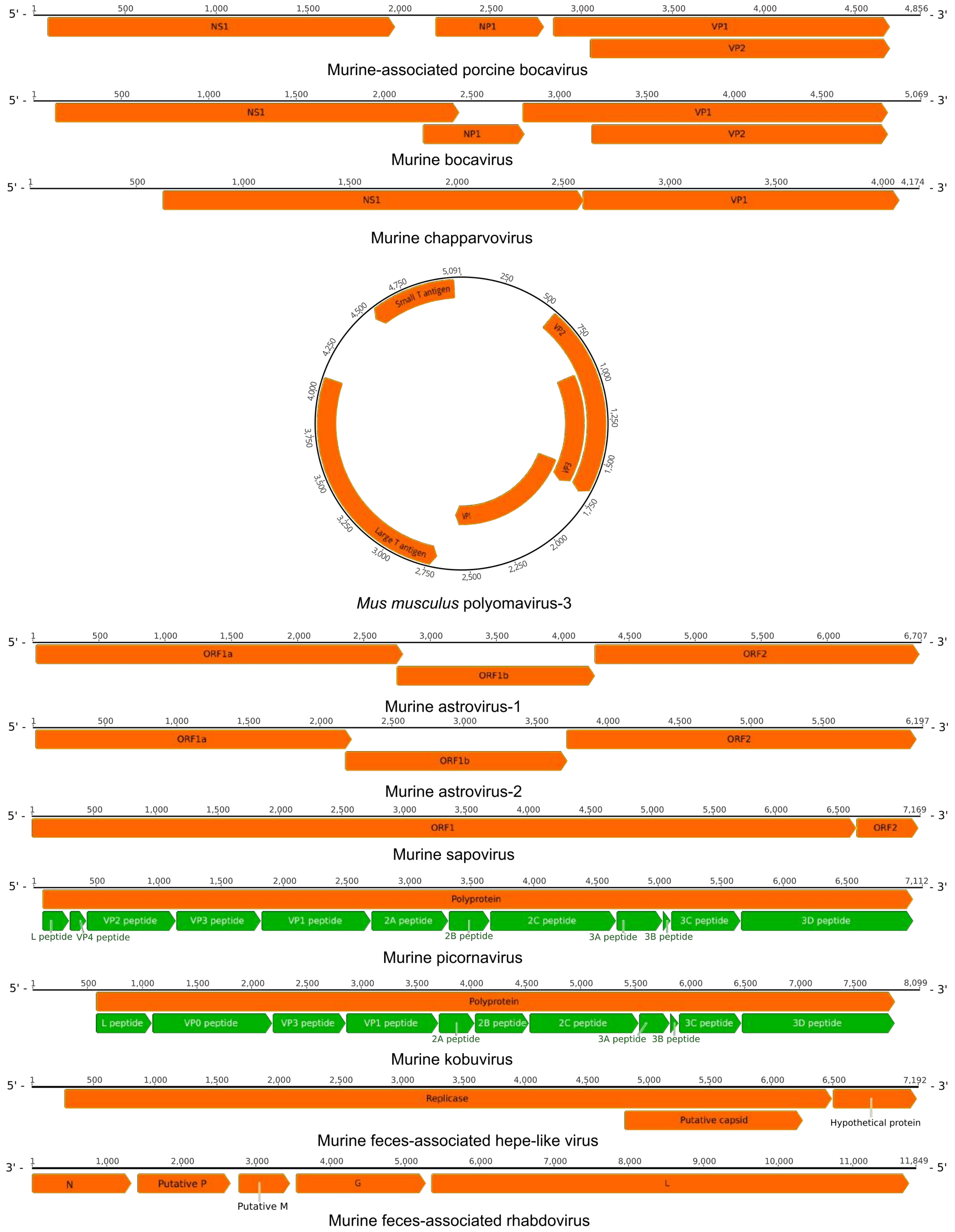

Supplement: FIG S2 [file mbo006173635sf2.pdf]
