# Supplementary material for: Viral Diversity of House Mice in New York City
Source: mBio. 2018 Apr 17;9(2):e01354-17. doi: 10.1128/mBio.01354-17 (PMC5904411; doi:10.1128/mBio.01354-17)

Fig. S3. Maximum likelihood phylogenetic tree of the large T antigen protein of viruses of the family *Polyomaviridae* (sequences obtained from (55)). The scale bar represents units of substitutions per site. The polyomavirus identified in this study and its associated genus is labeled in green. All other genera are labeled in a grey box. All bootstrap nodal support values are indicated if >70.

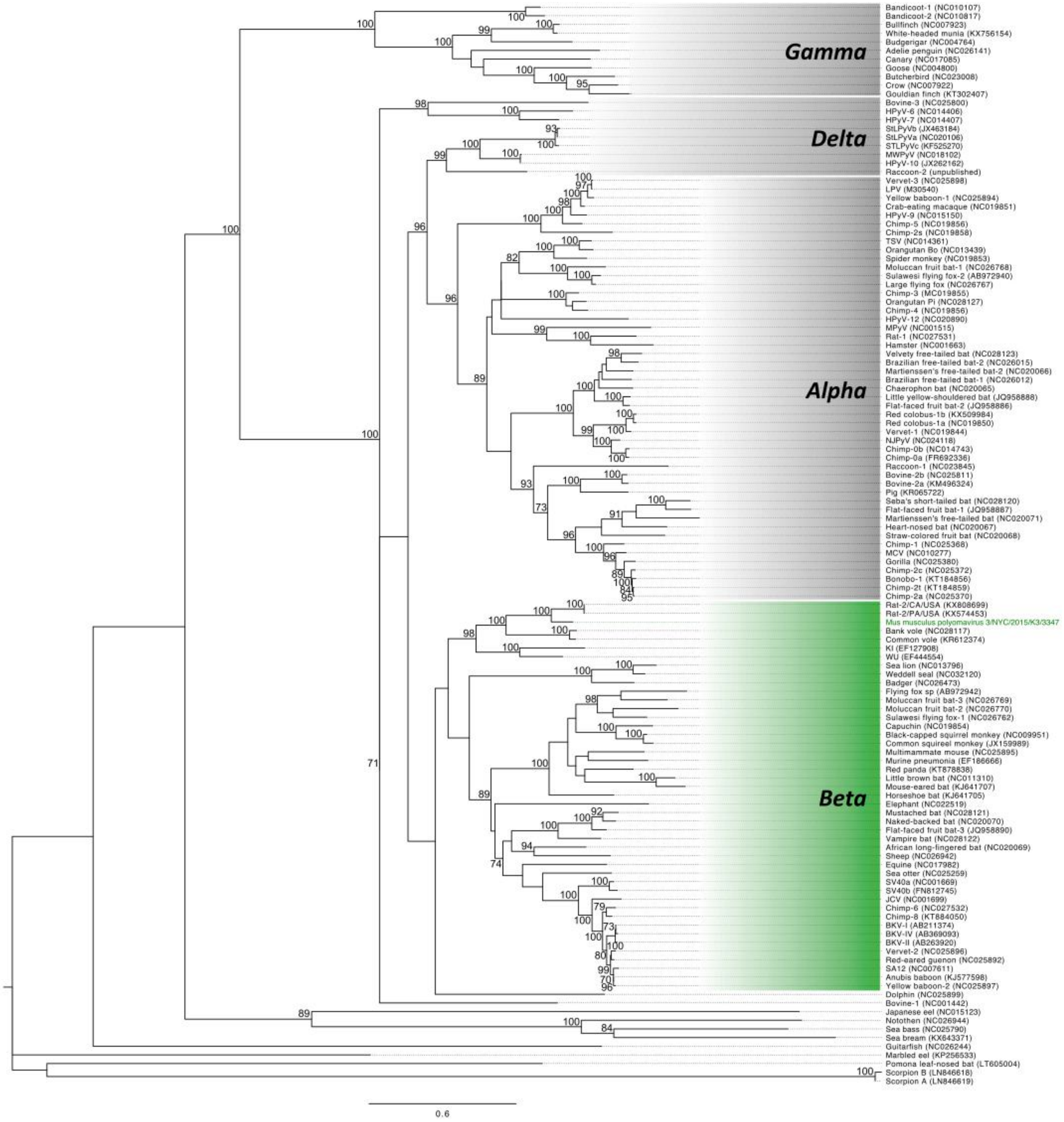

Supplement: FIG S3 [file mbo006173635sf3.pdf]
