# Supplementary material for: Viral Diversity of House Mice in New York City
Source: mBio. 2018 Apr 17;9(2):e01354-17. doi: 10.1128/mBio.01354-17 (PMC5904411; doi:10.1128/mBio.01354-17)

Fig. S4. Maximum likelihood phylogenetic tree of the capsid protein for representative members of the viral family *Astroviridae*. The scale bar represents units of substitutions per site. Genera are labeled with a black circle and recognized species are marked in grey boxes (MAstV, mammalian astrovirus; AAstV, avian astrovirus). Viruses identified in this study and the unclassified clades that they are assigned to are labeled in green. All bootstrap nodal support values are indicated if >70.

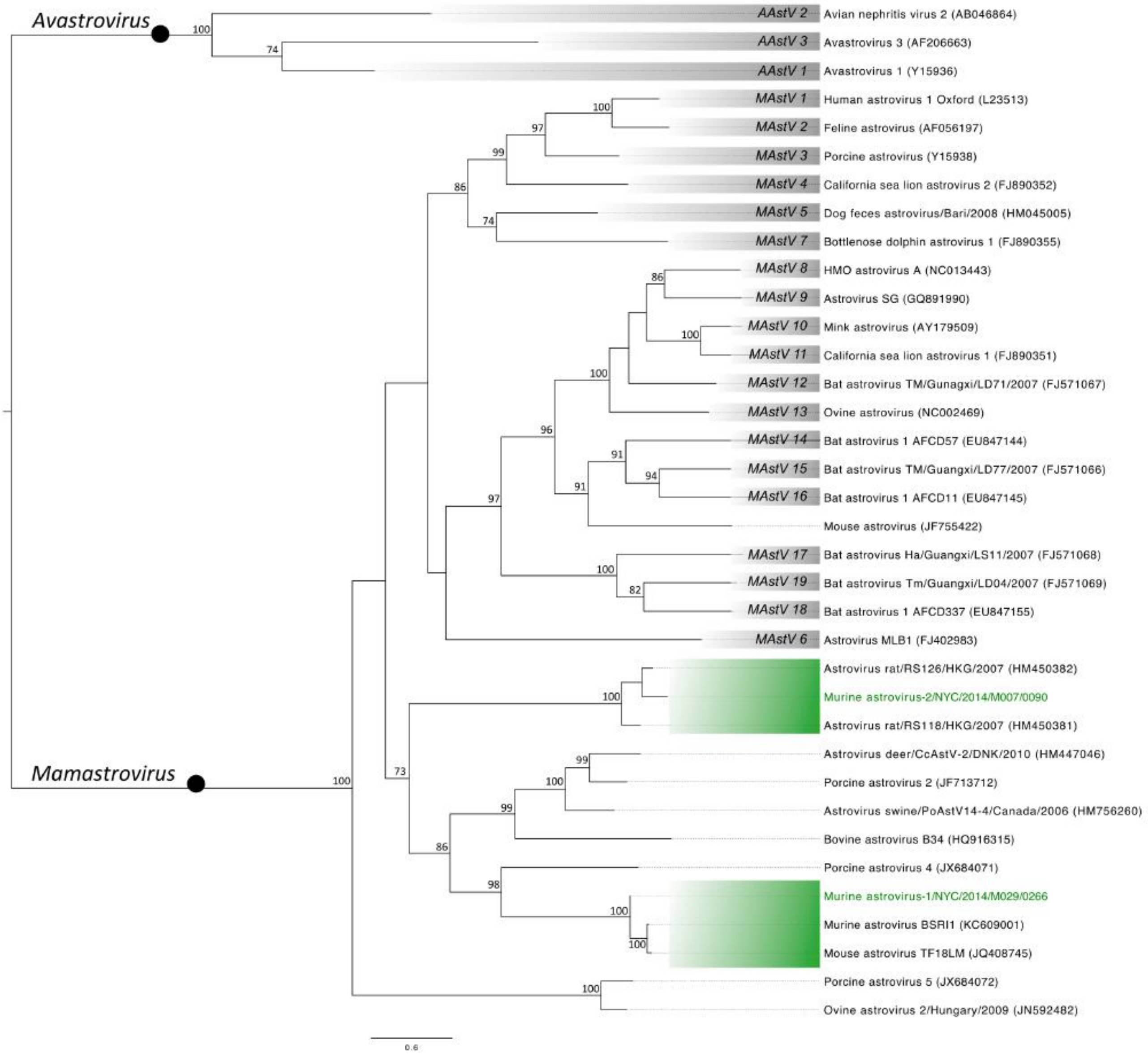

Supplement: FIG S4 [file mbo006173635sf4.pdf]
