# Supplementary material for: Viral Diversity of House Mice in New York City
Source: mBio. 2018 Apr 17;9(2):e01354-17. doi: 10.1128/mBio.01354-17 (PMC5904411; doi:10.1128/mBio.01354-17)

Fig. S5. Maximum likelihood phylogenetic tree of VP1 for members of the viral family *Caliciviridae*. The scale bar represents units of substitutions per site. Genera are labeled with a black circle. Representative viruses from currently recognized genogroups for the *Sapovirus* and *Norovirus* genera are shaded in grey boxes. Viruses identified in this study and the associated genogroups are labeled in green. All bootstrap nodal support values are indicated if >70.

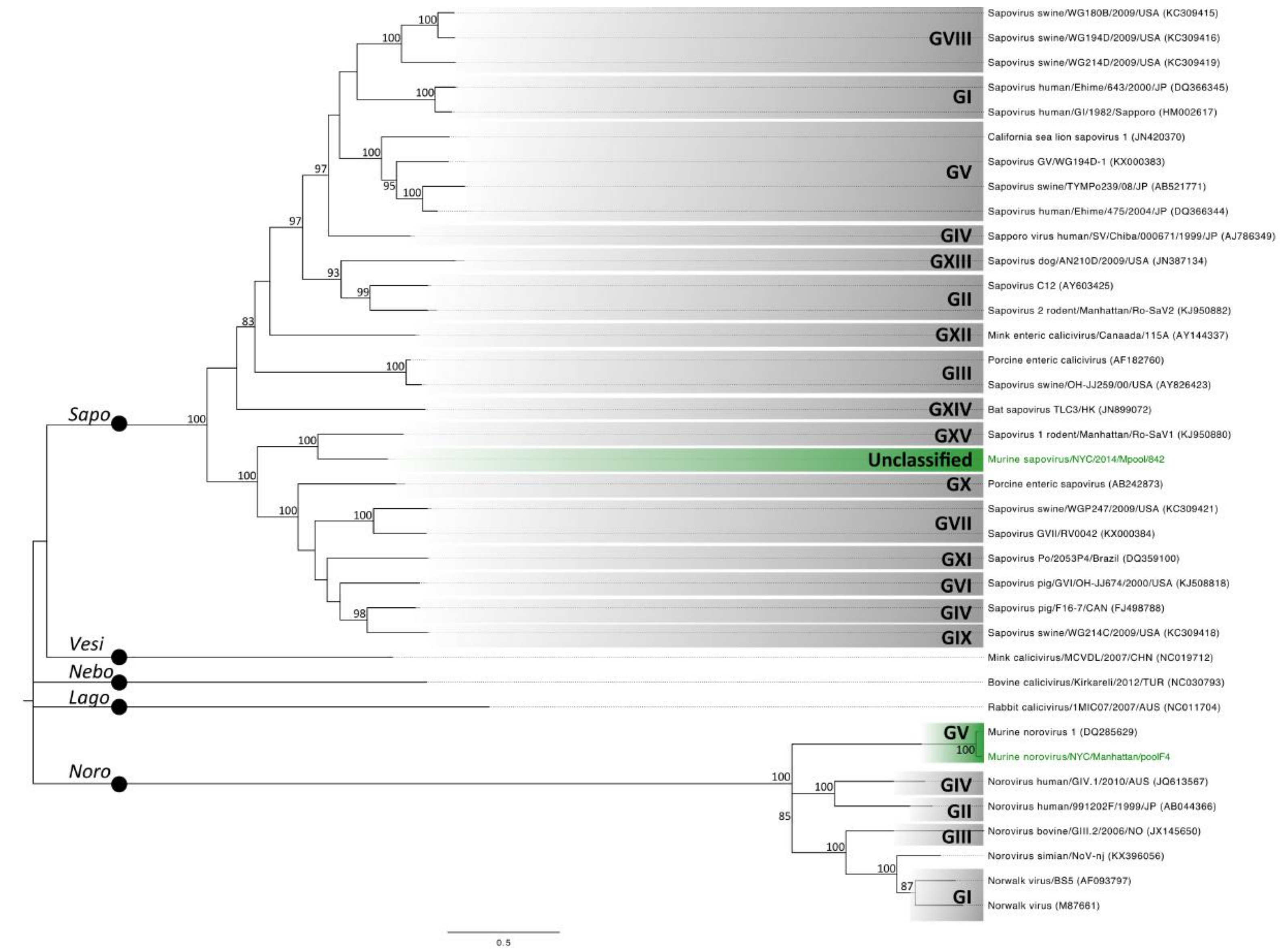

Supplement: FIG S5 [file mbo006173635sf5.pdf]
