# Supplementary material for: Viral Diversity of House Mice in New York City
Source: mBio. 2018 Apr 17;9(2):e01354-17. doi: 10.1128/mBio.01354-17 (PMC5904411; doi:10.1128/mBio.01354-17)

Fig. S6. Maximum likelihood phylogenetic tree of concatenated helicase/polymerase proteins for representative members of the *Hepe-Virga* clade. The scale bar represents units of substitutions per site. The hepe-like virus identified in this study and the clade that it is assigned to is labeled in green. All other recognized viral families are labeled in a grey box. All bootstrap nodal support values are indicated if >70.

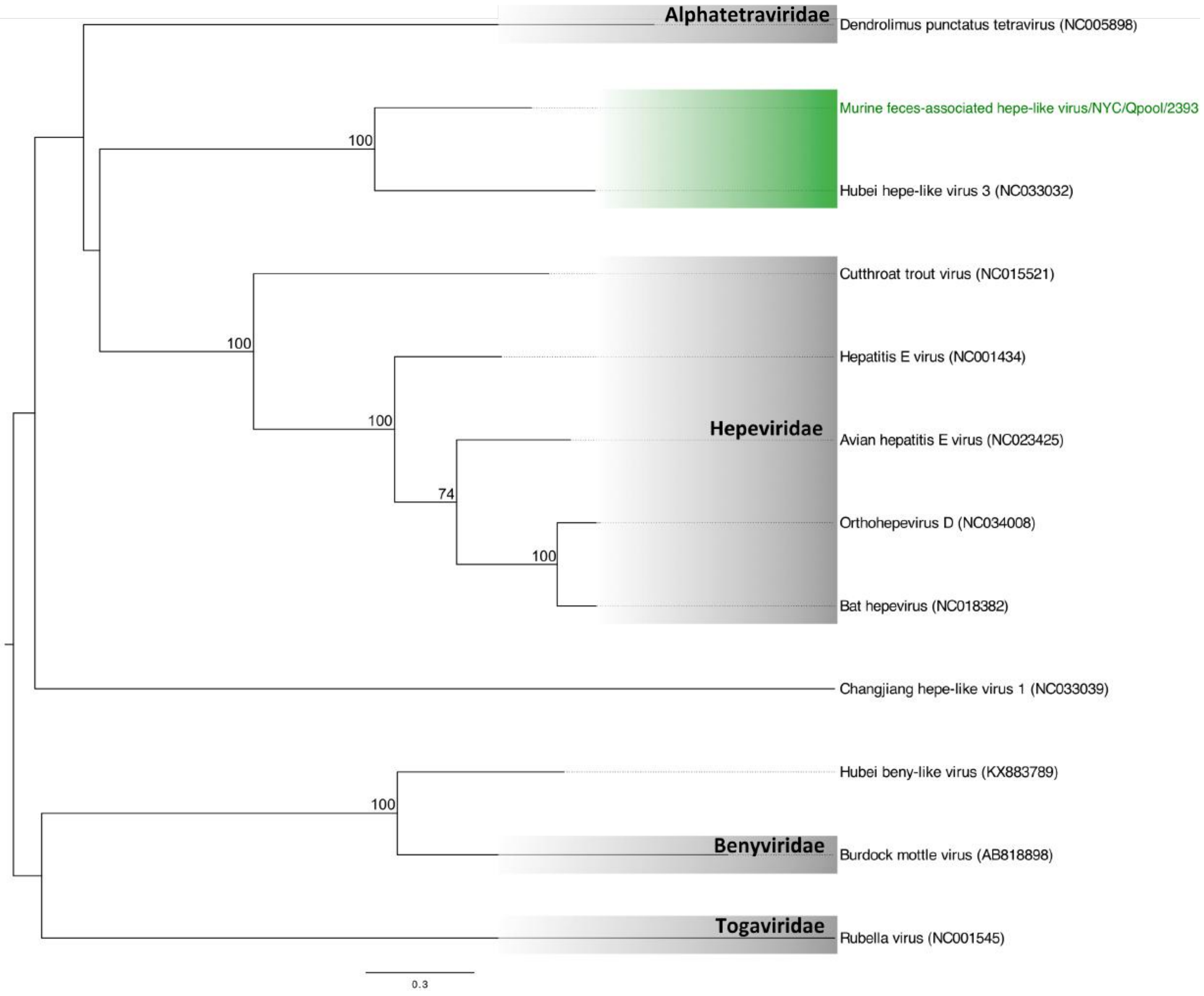

Supplement: FIG S6 [file mbo006173635sf6.pdf]
