# Supplementary material for: Viral Diversity of House Mice in New York City
Source: mBio. 2018 Apr 17;9(2):e01354-17. doi: 10.1128/mBio.01354-17 (PMC5904411; doi:10.1128/mBio.01354-17)
Supplement: FIG S7 [file mbo006173635sf7.pdf]

Fig. S7. Change in prevalence of fecal virus detections by PCR between two time points at two sites in NYC.

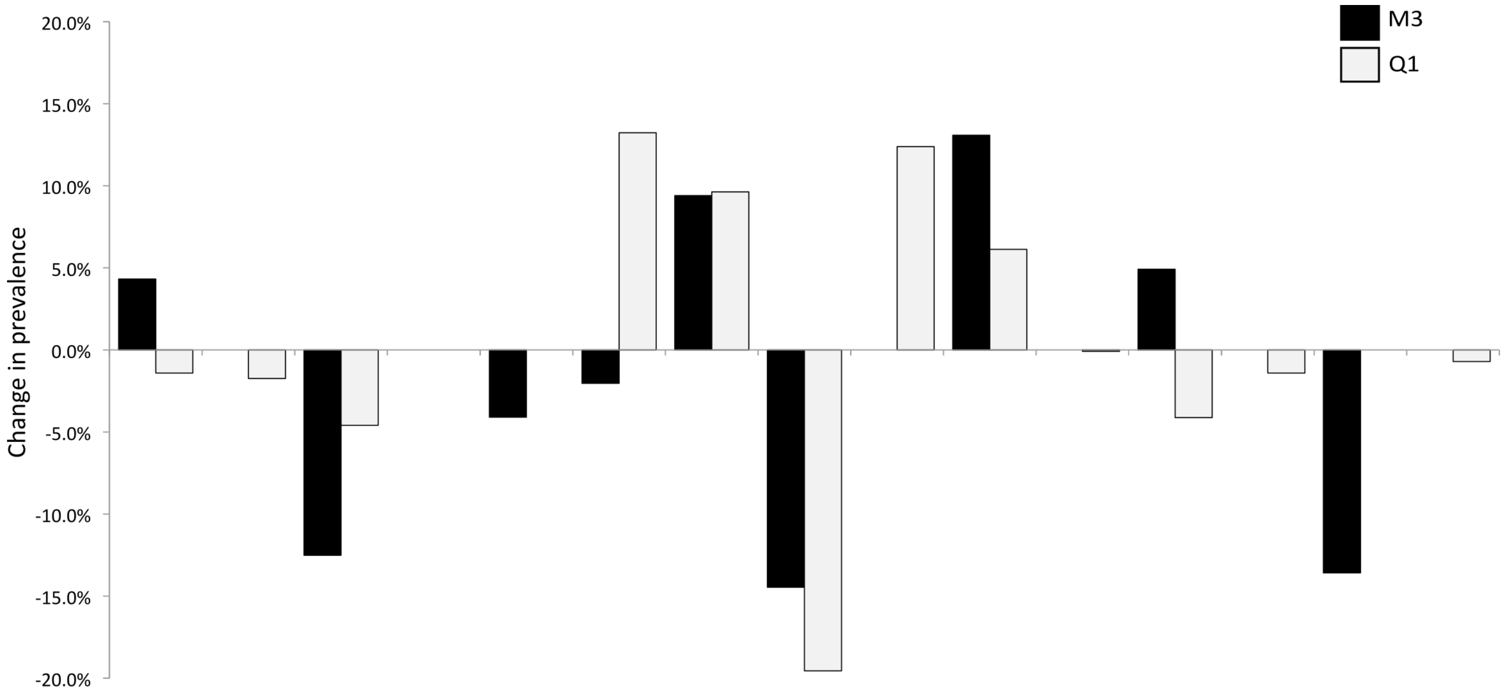

	MuAPBV	MuBV	MuCPV	MmusPyV-3	LaDV	MHV	MuAst-1	MuAst-2	MNV	MuSav	MuPiV	MuKoV	MuFAHLV	MuRotaV	MuFARV
M3-1	40.7%	0.0%	50.8%	0.0%	45.8%	22.0%	35.6%	67.8%	0.0%	15.3%	0.0%	5.1%	0.0%	15.3%	0.0%
M3-2	45.0%	0.0%	38.3%	0.0%	41.7%	20.0%	45.0%	53.3%	0.0%	28.3%	0.0%	10.0%	0.0%	1.7%	0.0%
Q1-1	1.4%	10.6%	45.8%	0.0%	0.0%	0.0%	18.3%	40.1%	14.1%	5.6%	14.8%	23.2%	1.4%	0.0%	0.7%
Q1-2	0.0%	8.8%	41.2%	0.0%	0.0%	13.2%	27.9%	20.6%	26.5%	11.8%	14.7%	19.1%	0.0%	0.0%	0.0%
