# Supplementary material for: Viral Diversity of House Mice in New York City
Source: mBio. 2018 Apr 17;9(2):e01354-17. doi: 10.1128/mBio.01354-17 (PMC5904411; doi:10.1128/mBio.01354-17)
Supplement: TABLE S1 [file mbo006173635st1.pdf]

Supplemental Table 1. Pairwise association between virus prevalence and site

Virus	Site pairwise comparison	OR	95% confidence interval Lower limit	Upper limit	p-value*
MuAPBV	M3 vs M2	54.4	7.1	7.0×10^{3}	2.1×10^{-7}
	M3 vs Q1	60.0	19.3	300.1	$<1.0 \times 10^{-11}$
	M3 vs X1	57.0	7.5	7.3×10^{3}	9.3×10^{-8}
MuBV	Q1 vs M3	28.6	3.7	3.7×10^{3}	8.5×10^{-5}
MuCPV	Q1 vs X1	5.9	2.4	17.4	5.5×10^{-5}
LaDV	M3 vs M2	9.6	2.7	51.1	1.5×10^{-4}
	M3 vs Q1	240.2	33.4	3.1×10^{4}	$<1.0 \times 10^{-11}$
	M3 vs X1	41.3	5.3	5.3×10^{3}	6.5×10^{-6}
MHV	M3 vs M2	47.3	5.8	6.2×10^{3}	6.5×10^{-6}
	M3 vs Q1	8.7	3.8	21.4	6.8×10^{-8}
	M3 vs X1	31.9	4.0	4.1×10^{3}	7.0×10^{-5}
MuAst-1	M3 vs Q1	2.6	1.6	4.4	2.7×10^{-4}
MuAst-2	M2 vs X1	69.2	8.3	9.1×10^{3}	5.5×10^{-7}
	M3 vs X1	103.4	13.6	1.3×10^{4}	5.0×10^{-11}
	Q1 vs X1	47.3	6.4	6.0×10^{3}	2.4×10^{-7}
MNV	M2 vs M3	141.0	16.6	1.9×10^{4}	4.4×10^{-9}
	M2 vs X1	37.7	4.4	5.0×10^{3}	9.4×10^{-5}
	Q1 vs M3	74.1	10.0	9.5×10^{3}	8.2×10^{-10}
MuSaV	M3 vs Q1	4.4	2.2	9.2	2.7×10^{-5}
	M3 vs X1	26.8	3.5	3.5×10^{3}	1.6×10^{-4}
MuPiV	Q1 vs M3	38.2	5.1	4.9×10^{3}	3.1×10^{-6}
MuKoV	Q1 vs X1	25.6	3.5	3.3×10^{3}	7.2×10^{-5}
MuRotaV	M3 vs Q1	42.7	5.2	5.5×10^{3}	2.4×10^{-5}

Odds ratios (OR) were calculated using Firth logistic regression analysis. Only statistically significant associations shown (family-wise error rate controlled at 0.05 level)
