# Supplementary material for: Viral Diversity of House Mice in New York City
Source: mBio. 2018 Apr 17;9(2):e01354-17. doi: 10.1128/mBio.01354-17 (PMC5904411; doi:10.1128/mBio.01354-17)
Supplement: TABLE S2 [file mbo006173635st2.pdf]

Supplemental Table 2. PCR primers and cycling conditions

Agent	Target gene	Forward primer (5'-3')	Reverse primer (5'-3')	Product size (nt)	AT (°C)	Reference
Viruses						
MuAPBV	NS1	ACAAGAGATTGCGGATTGGC	CTTACCTCCGTCGTCCCCAA	247	60-55	This study
MuBV	NS1	ATCTGGTGGGAGGAGTGTC	AACAGTCGACTAGCCAGTCG	298	60-55	This study
MuCPV	NS1	CCACTAATCATGCTCCCTGGC	ACCTTCTGCTGCAGGTAGTGG	317	60-55	This study
MmusPyV-3	VP1	GGTTACTGCTACAGGACTGAC	CTTGAAGGGTCAGGAATCCAG	246	60-55	This study
LaDV	Polyprotein–1b	TACCTGTTGAGTGTGGTCC	CCTCAATCAACTGTCTCCC	355	60-55	This study
MHV	Orf1b	GCGTTCTCTTGATAGAGCGC	GAACTACAGACAACGCAGGC	271	60-55	This study
MuAst-1	Orf1b/2	GAAACTCTTGAGCCTGCAGC	CAGTCCGAACAGTTGTTGTC	321	60-55	This study
MuAst-2	Orf1b/2	TCACCTGGAGGCTCTCTGG	CATTATTAGGCTGGACGCGC	238	61-56	This study
MNV	VP1	CACCTCTCAGCCATGTAYAC	AGTGGCGTGTACAGCATGC	287	60-55	This study
MuSaV	VP1	TCAGGGTCTCKGTSTCGG	CCCAGATGCCAAGRGTCTG	225	62-57	This study
MuPiV - M	VP1	CATCCAGGTACAGAGAGCC	GGTGTAGTGGGCATGTACC	342	62-57	This study
MuPiV - Q	VP1	GCCAACTTCATGTGCAAGC	GGTGTTGTSGGCATGTACC	371	62-57	This study
MuPiV - K	VP1	GCCAACTTCATGTGCAAGC	GGAGTAGTGGGCATGTACC	371	60-55	This study
MuKoV	Polyprotein-2b	TGACCGCTGACGCAGAGAC	YTTGGCRAGCCAGGTGAGG	269	60-55	This study
MuFAHLV - MQ	Replicase	AAGGARAGTAARTGTGCCC	TTTTGCTCCAYGCSGAGAC	349	58-53	This study
MuFAHLV - X	Replicase	AAGGAAAGTAAATGTGCCC	TTTTGCTCCACGCAGAGAC	349	57-52	This study
MuRotaV	VP1	TGGGATCAAGCTGCAGTGC	CCTGATAGAGACATGAACGCC	260	60-55	This study
MuFARV	L protein	TCTGGTGAAAGAGGAGGTC	TCCTTCTACTATCCCAGGG	196	60-55	This study
Arenavirus	L segment	AGAATYAGTGAAAGGGARAGYAAYTC	CACATCATTGGTCCCCATTTACTRTGATC	395	55	[1]
LCMV	S segment	CGCACAGTGGATCCTAGGC	CWAGRTCRGCYGCAARRGAC	380	64-59	This study
Housekeeping						
GAPDH		TGACGTGCCGCCTGGAGAAA	AGTGTAGCCCAAGATGCCCTTCAG	98	60*	[2]
M. musculus mitochondrial d-loop		TGAATTGGAGGACAACCAGT	TTATAAGGCCAGGACCAAAC	1240	58-53	[3]

AT, PCR annealing temperature; AT* listed as xx-yy indicates touchdown PCR (delta 0.5°C/cycle for 10 cycles);
*assay performed as a SYBR-green real-time PCR with 60s combined anneal and extension

1. Vieth, S., et al., *RT-PCR assay for detection of Lassa virus and related Old World arenaviruses targeting the L gene.* Trans R Soc Trop Med Hyg, 2007. **101**(12): p. 1253-64.
2. Mamo, S., et al., *Quantitative evaluation and selection of reference genes in mouse oocytes and embryos cultured in vivo and in vitro.* BMC Dev Biol, 2007. **7**: p. 14.
3. Gabriel, S.I., et al., *Of mice and 'convicts': origin of the Australian house mouse, Mus musculus.* PLoS One, 2011. **6**(12): p. e28622.
